# Effect of *Arthrospira platensis* (Spirulina) Fortification on Physicochemical, Nutritional, Bioactive, Textural, and Sensory Properties of Vegan Basil Pesto

**DOI:** 10.3390/nu16172825

**Published:** 2024-08-23

**Authors:** Izabela Podgórska-Kryszczuk

**Affiliations:** Department of Analysis and Food Quality Assessment, University of Life Sciences in Lublin, Skromna 8, 20-704 Lublin, Poland; izabela.podgorska-kryszczuk@up.lublin.pl

**Keywords:** *Arthrospira platensis*, spirulina, microalgae, basil pesto, antioxidants, food fortification, vegan, novel foods

## Abstract

The high protein content of several microalgae species makes them an excellent addition to various food products, increasing their nutritional value. In this study, vegan basil pesto was designed and enriched with 1% and 2% *Arthrospira platensis* (spirulina). The pesto obtained was characterized by increased protein content (up to 40% more) and had a rich mineral composition, including up to three times more iron and 25% more calcium, among others. The increase of spirulina addition in the pesto also increased the content of polyphenols (up to 50% more) and flavonoids (up to 39% more). The fortified products had higher antioxidant activity against ABTS (up to 484.56 ± 2.16 μM Trolox/g) and DPPH (up to 392.41 ± 13.58 μM Trolox/g). The addition of spirulina will affect the hardness of the sauce, while in the other texture parameters (adhesiveness, springiness, and cohesion), there were no significant differences between the control and spirulina-fortified pesto. Although the pesto with spirulina was significantly darker in color (ΔE 8.83 and 12.05), consumers still rated it highly. All quality parameters of pesto with a 1% spirulina addition were rated the highest, contributing to the highest overall rating of the product (4.56). An increase in spirulina addition to 2% resulted in a decrease in the overall pesto rating (4.01), but still remains a good result compared to the control (4.22).

## 1. Introduction

Basil pesto sauce is commonly used as a dressing for pasta and ranks second in popularity after tomato sauce. The traditional Italian sauce is made from fresh basil leaves, garlic, cheese, and pine nuts, adding olive oil. Pesto is extremely popular among consumers, not only for its distinctive flavor and aroma but also for its special color [1,2]. Unfortunately, due to the low stability and sensitivity of pesto sauce to the impact of environmental factors, an enzymatic browning reaction can occur, which negatively affects the sensory characteristics of the product. This process in vegetable products can be delayed by adding antioxidant substances [3]. Moreover, modern consumers pay more attention to food composition, functionality, and its health-promoting nature, and avoid using preservatives or synthetic colors. To meet consumer expectations and increase product competitiveness, food manufacturers are forced to expand the range of products characterized not only by taste and desirable sensory qualities but also by high nutritional value and pro-health properties [1]. High-quality foods with enriched nutritional, mineral, and bioactive properties can be produced from microalgae [4,5]. Microalgae, including spirulina, are now sold as dietary supplements and are widely used to design new food products [6]. The term spirulina is used to describe mainly two cyanobacteria species: *Spirulina platensis* and *Spirulina maxima*. According to the United States Food and Drug Administration (FDA), spirulina is classified as “Generally Recognized As Safe” (GRAS) when used properly [7]. Spirulina has been incorporated into several food products, such as pasta [8,9], bread [10], kefir [5], yogurt [11], ayran [12], Ricotta cheese [13], ice cream [14], biscuits [15], chocolate milk [16], and chicken mortadella [17]. This is a result of the rich composition of spirulina. Particular attention should be paid to its high protein content with a well-balanced amino acid profile [18]. Spirulina is also a source of polyunsaturated fatty acids (PUFA), such as eicosapentaenoic acid (EPA) and docosahexaenoic acid (DHA) [6]. In addition, microalgae are rich in trace elements (sodium, potassium, iron, calcium, magnesium, zinc, and phosphorus) [15] and vitamins (vitamin B1, B2, B3, B6, B9, B12, C, D, and E) [18]. Spirulina also contains large amounts of carotenoids (astaxanthin, zeaxanthin, and β-carotene), polyphenols, and chlorophyll. Because of its rich composition, microalgae have antioxidant, anti-inflammatory and immunomodulatory properties [6]. The beneficial effects of spirulina in the treatment of cardiovascular diseases have been proven [19]. It has been shown to effectively lower triacylglycerols and low-density lipoprotein (LDL) cholesterol levels and help lower blood pressure [20]. In addition, it has been reported to have a blood glucose-lowering effect on people with diabetes and to aid weight loss [21]. Through beneficial antioxidant and anti-inflammatory effects, spirulina has also been shown to affect glial cell activation and prevent neurodegenerative diseases, particularly Alzheimer’s and Parkinson’s disease and multiple sclerosis [7]. Spirulina is especially appreciated by vegans and vegetarians, who risk numerous deficiencies, including high-quality protein with all the essential amino acids. Its mineral composition (exceptionally high iron and calcium content) makes it ideal for supplementing plant-based diets [6,15,21].

The purpose of the present study was to design a functional product: a vegan basil pesto with spirulina (1% and 2%), and to investigate the effect of the microalgae addition on the quality of the sauce—its physicochemical, nutritional, antioxidant, textural, and organoleptic properties. Due to the rich composition of spirulina, the newly created pesto can be an extremely competitive product, enriching the diet of vegans and vegetarians in particular.

## 2. Materials and Methods

### 2.1. Chemicals

Trolox (6-hydroxy-2,5,7,8-tetramethylchroman-2-carboxylic acid), DPPH (2,2-diphenyl-1-picrylhydrazyl), ABTS (2,20-azino-bis(3-ethylbenzothiazoline-6-sulfonic acid)), Folin–Ciocalteu reagent (FCR), gallic acid, epicatechin, α-amylase, pancreatin, pepsin, and bile extract were purchased from Sigma-Aldrich Company, St. Louis, MO, USA. Standard solutions for each element (calcium, magnesium, potassium, iron, and phosphorus) were obtained from Merck Millipore (Burlington, MA, USA). All other chemicals were of analytical grade.

### 2.2. Pesto Preparation

Basil pesto was prepared in three variations: control pesto without added spirulina (BP0), pesto with 1% added spirulina (BP1), and pesto with 2% added spirulina (BP2). In the BP1 and BP2 cases, spirulina partially replaced fresh basil. Fresh basil was purchased from a local supermarket, while all other ingredients were purchased from an online mail-order store (MarketBio.pl, Wroclaw, Poland). The study used spirulina microalgae powder with organic certification (Batom.pl, Cracow, Poland). The ingredients were weighed according to the recipe shown in Table 1 and then placed in a cup blender pitcher (Philips, Amsterdam, The Netherlands). Each sample was blended until homogeneous for about 5 min. Samples for determination were kept refrigerated for up to 48 h after preparation. Parts of the sample were frozen (−20 °C, 48 h) and then lyophilized (Delta 2-24 LSCplus, Christ, Osterode am Harz, Germany) under the following parameters: drying chamber temperature at −65 °C, shelf temperature at 20 °C, pressure at 0.5 mbar (48 h).

### 2.3. Color and pH Measurement

The color of pesto sauces was evaluated by the instrumental method of the CIE L*a*b color model using a colorimeter (NH310, EnviSense, Lublin, Poland). The measuring instrument was calibrated with a white standard. Yellow/blue (b*), green/red (a*), and lightness (L*) measurements were made. The total color difference (ΔE) was calculated using the following formula:

$$∆E=\sqrt{{∆L}^{*2}+{∆a}^{*2}+{∆b}^{*2}}$$

where: ΔL*, Δa*, and Δb* are differences in the L*, a*, and b* values, respectively, between the reference sample and the test sample.

The pH in the prepared pesto was measured using the potentiometric method with a laboratory pH meter probe (Elmetron CP-511, Elmetron G.P., Zabrze, Poland) at room temperature (20 °C).

### 2.4. Nutrient Composition

Using standard analysis methods, the pesto samples were evaluated for moisture, ash, protein (N × 6.25), and fat content [22]. The moisture content was gravimetrically determined by drying the pesto samples in an oven (SUP-65W, Wamed, Warsaw, Poland) at 120 °C until a constant weight was reached. Crude fat was determined using the Soxhlet extraction method (Tecator Soxtec System HT 1043 extraction unit, Gemini, Apeldoorn, Sweden), which used hexane as the extracting agent. Crude protein content (N × 6.25) was determined using the Kjeldahl method in the automatic distiller Kjeltec 8400 (Foss, Foss Analytical AB, Höganäs, Sweden). The ash content was determined by incinerating the pesto samples in a muffle furnace FCF S (Czylok, Jastrzębie Zdrój, Poland) at 900 °C for 60 min. Carbohydrate content was determined using the following formula: 100 − [weight in grams (moisture + protein + fat + ash) in 100 g of fresh pesto] [23]. The conversion method determined the nutritional value; the following conversion factors were used: 9 × (g fat) and 4 × (g protein + g carbohydrate) [23].

### 2.5. In Vitro Protein Digestibility and Protein Availability

*In vitro* protein digestibility was assessed by the total soluble protein content, and protein content was determined after *in vitro* digestion [9]:

PD [%] = [(Pt − Pd) × 100%]/Pt

where PD represents the *in vitro* protein digestibility; Pt represents the total soluble protein content; and Pd represents the protein content after *in vitro* digestion.

Protein availability was calculated based on the protein content of the pesto and protein digestibility [9]:

PA [g/100 g] = PD × Pt/100%

where PA represents protein availability; PD represents the *in vitro* protein digestibility; and Pt represents the total soluble protein content.

#### *In Vitro* Digestion

*In vitro* digestion was carried out according to the methodology of Zielińska et al. [24]. Briefly, the lyophilized pesto was ground in a laboratory grinder (IKA A11 basic, IKA-Werke GmbH & Co. KG, Staufen, Germany). A stimulated saliva solution (7 mM NaHCO_3_ and 0.35 mM NaCl) was prepared and adjusted to pH = 6.75. Sample solutions (4%, *w*/*v*) were incubated in stimulated saliva for 5 min at 37 °C. Hydrolysis followed this with α-amylase (50 U/mg, enzyme to substrate ratio was 1:10; *w*/*v*) for 10 min at 37 °C. Under simulated gastric digestion conditions (pH = 2.5, 2 h, 37 °C), samples were hydrolyzed with pepsin (250 U/mg, enzyme to substrate ratio was 1:100; *w*/*w*). The solution was adjusted to pH 7.0 with 1 M NaOH for simulated intestinal juice. A mixture with 0.7% pancreatin solution and 2.5% bile extract solution (1:2.5; *v*/*v*) was added, and the process was carried out at 37 °C for 1 h. The samples were heated at 100 °C for 5 min to inactivate the enzymes. The entire process took place in the dark. The final step was centrifuging the hydrolysates at 8000 rpm for 10 min. The supernatants were separated and used for further analysis.

### 2.6. Minerals Content

To determine calcium, magnesium, potassium, and iron content, 0.5 g of each lyophilized pesto sample was weighed, and 4 mL of nitric acid (V) was added. Then, the samples were mineralized in a microwave oven (Mars, Xpress, CEM Corporation, Matthews, NC, USA). The minerals were quantitatively transferred to 25 mL volumetric flasks which were filled up to the mark with deionized water. The concentration of mineral ions in the minerals was determined by flame atomic absorption spectrophotometry (FAAS, Solaar 939, Unicam, Ilminster, UK) at 422.7 nm for calcium, 285.2 nm for magnesium, 766.5 nm for potassium, and 248.3 nm for iron using an air acetylene flame [25].

To determine phosphorus content, the lyophilized pesto sample was ashed with calcium carbonate and heated with hydrochloric acid and nitric acid (V). Part of the acidic solution was mixed with molybdovanadate reagent, and the absorbance of the resulting solution was measured spectrophotometrically (Cary 50 Scan, Varian, Palo Alto, CA, USA) at 430 nm [26].

### 2.7. Bioactive Properties

#### 2.7.1. Bioactive Compounds Extraction

From each pesto sample, 1 g was taken and transferred to Falcon tubes. Then, 10 mL of solvent (ethanol/water) was added in a 4:1 (*v*/*v*) ratio and shaken in a laboratory shaker (Multi Bio RS-24, Biosan, Riga, Latvia) for 2 h at 150 rpm at room temperature. After this time, the samples were centrifuged at 3000 rpm for 10 min. The extract was stored under freezing conditions (−20 °C) until further analysis. 

#### 2.7.2. Total Polyphenol Content (TPC)

The study was performed according to the methodology of De Bruno et al. [1], slightly modified for the study material. Briefly, 0.04 mL of the prepared extract, 3.16 mL of distilled water, and 0.2 mL of FCR were added to the tubes and mixed. After 3 min, 0.6 mL of sodium carbonate solution (100 g/L) was added. The tubes were left in a thermostat set to 40 °C for 30 min. After the indicated time, the absorbance of the test pesto samples was measured using a UV–Vis spectrophotometer at 765 nm against a blank sample. The results were expressed as gallic acid equivalent per 100 g of pesto sample (mg GAE/100 g). 

#### 2.7.3. Total Flavonoids Content (TFC)

TFC was determined spectrophotometrically, according to the method of Karadeniz et al. [27]. In a 10 mL volumetric flask, 1 mL of prepared extract, 5 mL of distilled water, and 0.3 mL of a 5% aqueous solution of sodium nitrate (III) were measured. The resulting solution was stirred on a vortex and allowed to stand for 5 min. After this, 0.6 mL of a 10% aqueous solution of aluminum chloride hexahydrate was added, remixed, and left for 5 min. Then, 2 mL of sodium hydroxide solution (1 M) was added and filled with distilled water to the measuring line. The absorbance of the prepared samples was measured at 510 nm against the blank. TFC was expressed as the amount of epicatechin in 100 g of pesto sample (mg EPI/100 g).

#### 2.7.4. DPPH Radical Scavenging Activity

The determination was performed according to the methodology of Baliyan et al. [28] and slightly modified. A total of 0.1 mL of the prepared extract was added to 2.9 mL of a 6 µM DDPH solution in 75% methanol, and the mixture was vortexed. The tubes were stored at room temperature in a darkened place for 30 min. Then, the absorbance was measured at 517 nm against a blank sample. The results were expressed as Trolox equivalent antioxidant activity (TEAC) values (µM Trolox/g).

#### 2.7.5. ABTS Radical Scavenging Activity

The determination was performed according to the methodology of Re et al. [29] with slight modifications. The radical solution was prepared from ABTS and potassium persulfate, diluted in distilled water to a concentration of 2.45 mM and left in the dark for 16 h to allow the radical to develop. Then, the solution was diluted to give an absorbance of about 0.7 at 734 nm. In test tubes, 2.9 mL of ABTS solution was mixed with 0.1 mL of prepared extract. The tubes were set in a darkened place for 30 min, and then the absorbance at 734 nm against a blank sample was measured. The results were expressed as Trolox equivalent antioxidant activity (TEAC) values (µM Trolox/g).

### 2.8. Texture Profile Analysis (TPA)

The hardness, adhesiveness, springiness, and cohesion of the pesto were determined using a TA-XT2i texture analyzer (Stable Micro Systems, Godalming, UK). The obtained samples stored in cylindrical beakers were tested using a cylindrical sampler with a diameter of 10 mm at a head travel speed of 1 mm/s (the degree of immersion of the sampler was 50%, the interval between sampler movements was 5 s) at 21 °C [30]. The obtained results were recorded using the computer program Texture Expert version 1.22.

### 2.9. Semi-Consumer Evaluation

Using the five-point method, a semi-consumer evaluation of the prepared pesto samples was conducted with 35 people aged 21–45 (23 women and 12 men). The prepared pesto was separated into portions and coded with a three-digit number. Respondents rated the quality parameters of the pesto sauces. Each parameter was assigned a specific weighting factor: overall appearance (0.25), color (0.15), taste (0.3), aroma (0.1), and consistency (0.2). In the experiment, it was assumed that a score between 4.5 and 5.0 meant very high quality; a grade between 3.5 and 4.49 signified high quality; a grade between 2.5 and 3.49 signified moderate quality; a grade between 1.5 and 2.49 signified low quality; and a grade less than 1.49 signified very low quality [31]. Mineral water was used to rinse the mouth before each test. 

All respondents gave informed consent to participate in the study, which was approved by the University Ethical Committee for Research with Human Participation of the University of Life Sciences in Lublin (resolution number: UKE/27/2024).

### 2.10. Statistical Analysis

All assays were performed in at least three replicates, and the results are presented as mean ± SD (standard deviation). The results were analyzed statistically using Statistica (version 13.3, StatSoft, Cracow, Poland) for the comparison of means using a one-way analysis of variance (ANOVA) with a post hoc Tukey’s honestly significant difference (HSD) test. Statistical hypotheses were verified at the significance level of *p* < 0.05.

## 3. Results

### 3.1. Color and pH Measurements

**Figure 1:**
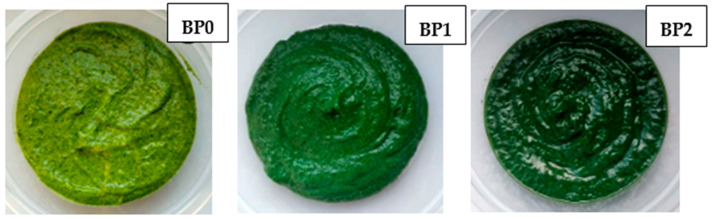
Appearance of prepared pesto samples. BP0—control basil pesto, BP1—basil pesto with 1% addition of spirulina, BP2—basil pesto with 2% addition of spirulina.

The general appearance of the prepared pesto samples is shown in Figure 1. The measured and calculated color parameters of the tested pesto samples are shown in Table 2. The BP0 sample had the brightest color (L* = 43.03 ± 0.58), while the color became significantly darker with the increased addition of spirulina to the pesto. Thus, the L* parameter values for the BP1 and BP2 samples were 36.92 ± 0.39 and 34.77 ± 0.83, respectively. The results of the a* parameter indicated a green coloration of all pesto samples, not statistically significantly different (from −5.99 ± 0.24 to −5.75 ± 0.33). Parameter b* showed that the higher the spirulina addition, the more intense the blue color in the final product. For this parameter, statistically significant differences were observed between the samples, and the highest value of the b* parameter was noted in the BP0 pesto (19.57 ± 0.32), and the lowest in the BP2 (10.80 ± 0.19). The parameter of total color difference ΔE indicated that the control and spirulina-enriched samples had two different colors for the consumer. The color difference was most noticeable for the 2% addition of spirulina to the pesto sauce and was ΔE = 12.05.

The addition of spirulina to the pesto resulted in a statistically significant increase in the product’s pH. The pH value in the BP0 sample was 4.88 ± 0.01 c, while in the BP1 samples, it was 4.95 ± 0.01 b, and in BP2, it was 4.99 ± 0.01 a.

### 3.2. Nutritional Value

Table 3 shows the nutritional value of the tested pesto samples. The spirulina addition significantly increased the protein content of the pesto compared to the control sample. There was a nearly 35% increase in protein for the BP1 sample and more than 40% for BP2. The protein content was not statistically significantly different between the variants with 1% and 2% spirulina additions. At the same time, the carbohydrate content was found to be lower in samples with the algae (BP1: 6.67 ± 0.52 g/100 g and BP2: 6.97 ± 0.43 g/100 g) compared to pesto without the additive used (9.89 ± 0.47 g/100 g). All prepared basil pesto samples did not statistically differ significantly in fat, ash, moisture content, and energy value.

### 3.3. In Vitro Protein Digestibility

Table 4 shows the *in vitro* protein digestibility results in fresh basil pesto and protein availability after digestion. The study found no significant differences in the protein digestibility of control and spirulina-enriched pesto. In contrast, significant differences were noted in the amount of available protein after digestion. The samples with spirulina showed higher protein availability compared to the control. For BP1, the PA was 6.88 ± 0.29, and for pesto BP2, it was 7.11 ± 0.02.

### 3.4. Minerals Content

Table 5 shows the mineral content of the control and spirulina-enriched pesto samples. The calcium (Ca) content of both samples with spirulina was about 25% higher than the control pesto. The amount of Ca in the enriched samples was similar: 97.64 ± 0.65 mg/100 g for BP1 and 98.47 ± 3.84 mg/100 g for BP2. The study found that the highest magnesium (Mg) and phosphorus (P) contents were in the pesto with 2% spirulina addition, 91.63 ± 0.44 mg/100 g and 234.46 ± 2.62 mg/100 g, respectively. There were no statistically significant differences noted in Mg and P content between the control sample and the sample with 1% algae addition. Potassium (K) and iron (Fe) contents increased with increasing spirulina addition, so the highest content of these elements was recorded in the BP2 sample. Iron showed the highest increase of all the elements determined, comparing the control sample with test samples BP1 and BP2. Compared to BP0 (1.98 ± 0.09 mg/100 g), the Fe content of the sample BP1 (4.09 ± 0.12 mg/100 g) increased twofold, while that of the sample BP2 (6.15 ± 0.07 mg/100 g) increased as much as threefold.

### 3.5. Bioactive Properties

#### Total Polyphenol Content, Total Flavonoids Content, and Antioxidant Activity

Table 6 shows the contents of phenolic compounds, flavonoids, and antioxidant activity in the tested pesto samples. The experiments show that TPC, TFC, and antioxidant activity in the tested pesto increased with the addition of spirulina. TPCs for BP1 and BP2 samples were 206.32 ± 6.34 mg GAE/100 g and 259.24 ± 9.30 mg GAE/100 g, respectively. Compared to the control, the content of phenolic compounds in the BP1 sample increased by 19%, while in the BP2 sample, it increased by almost 50%. The TFCs for the BP1 and BP2 samples were 24.82 ± 0.28 mg EPI/100 g and 29.88 ± 0.8 mg EPI/100 g, respectively. The flavonoid content of the pesto with 1% and 2% additions was 15% and 39% higher, respectively, than in the sample without the addition. The highest values of antiradical activity against both ABTS and DPPH were observed for the BP2 sample (484.56 and 392.41 µM Trolox/g, respectively). In addition, the experiment observed higher free radical scavenging activity for ABTS than DPPH.

### 3.6. TPA

The texture parameters, such as hardness, adhesiveness, cohesion, and springiness of the obtained pesto sauces, are shown in Table 7. The study found that the hardness parameter of the pesto increases with the addition of spirulina. Accordingly, the BP2 sample had the highest hardness (83.62 ± 1.14 N) and the BP0 sample had the lowest (70.46 ± 1.50 N). As for the other texture characteristics: adhesiveness, springiness, and cohesion, no significant differences were found between all pesto sauces.

### 3.7. Semi-Consumer Evaluation

The results of the semi-consumer evaluation of pesto using the five-point method are presented in Table 8. According to consumers, the pesto with 1% spirulina addition had the highest rating for all quality parameters (overall appearance 4.77, color 4.49, taste 4.57, aroma 4.26, and consistency 4.49). This sample also received the highest overall rating of 4.56 (very high quality). The 2% spirulina addition pesto received the lowest overall rating (4.01). However, it remained of high quality, and the results of individual distinguishing factors were not statistically significantly different from the control pesto. According to consumers, all three pesto samples did not differ in aroma.

## 4. Discussion

Food consumption patterns have changed dramatically over the past few years due to increased awareness of healthier living, an aging population, and people’s tendency to reduce meat consumption [32]. The popularity of plant-based diets is growing due to their lower environmental impact compared to diets containing meat. At the same time, plant-based diets are associated with improved human health and animal welfare. Non-animal food products are increasingly consumed, creating a business opportunity for the food industry [33]. In this study, a traditional recipe was modified, and a vegan pesto sauce with spirulina was designed. Inactive yeast flakes, used in vegan products as a thickener for soups and sauces and as an addition to pasta and vegetable dishes, were used as a substitute for the traditionally used Parmesan cheese. Spirulina (1% and 2%) was added to enrich the resulting sauce, which is a valuable source of protein, polyunsaturated fatty acids, vitamins, minerals, and antioxidants [6].

Spirulina, as an alkaline food (pH 6.82–6.93) [13,34], increased the pH of the resulting pesto (from pH 4.88 to pH 4.99). Incorporating spirulina into the diet can be a convenient solution to the pH problems of most traditional diets. Modern consumers who consume soft drinks, meat (beef, pork, or poultry), cheese, or eggs in excess are acidifying their bodies (pH < 7). Acidification of the body can cause many diseases, such as gout, diabetes, hypertension, heart disease, rheumatism, and cancer. In contrast, including more alkaline foods in the diet is associated with improved mental function, proper immune system function, kidney health, and higher energy levels [34,35]. 

The resulting sauce with spirulina had an increased total protein content compared to the unenriched pesto. This is explained by the high protein content of spirulina, which can be as high as 70%. Such protein is of high biological value, as it contains all essential amino acids in the ratios recommended by the Food and Agriculture Organization of the United Nations (FAO) [36]. Spirulina peptides have proven to exhibit antioxidant, anti-inflammatory, anticancer, antiatherosclerotic and antimicrobial effects [37]. Spirulina protein has good digestibility, making the alga a possible alternative protein source. Because of its good digestibility, the experiment found that the amount of available protein was significantly higher in pesto with spirulina. In general, the bioavailability of spirulina nutrients may be higher than other plant sources due to the cell wall structure, which, unlike indigestible cellulose, is composed of protein, carbohydrates, and fat [38]. In addition, other researchers have shown that proteins from microalgae have promising techno-functional features and are used as emulsifying, foaming, and gelling agents [39]. Designing a vegan sauce with a higher protein content than its traditional counterpart makes perfect sense, as vegans are particularly vulnerable to nutrient deficiencies, particularly protein [40]. The pesto with spirulina had altered protein and carbohydrate ratios compared to the control, but this did not significantly affect the sauce’s energy values. The reason is that protein and carbohydrates have the same conversion factor values as energy values. A significant difference in fat content can only cause a difference in energy value, which the study did not find. 

The pesto with spirulina obtained in the experiment was characterized by an increased content of selected minerals, which, as mentioned, is due to the alga’s rich mineral composition [15]. Particularly important is the increase in the iron content of the resulting sauce. Iron deficiency, one of the world’s most significant nutritional problems, can be caused not only by iron-poor foods but also by low iron availability in the diet. Vegans and vegetarians, children aged 0–5 years, and pregnant women are particularly at risk of iron deficiency and related anemia [41]. Notably, the spirulina-enriched pesto was found to have about a 25% increase in calcium content. This element is a building block of bones, teeth, and connective tissue. It participates in blood clotting, nerve impulse conduction, and the regulation of several hormones. Compounds formed with phosphorus are responsible for the typical structure of bones [6]. The results obtained in this study are consistent with reports by other authors, who also confirm the significant effect of spirulina on increasing the mineral content of enriched products [13,17]. 

The main ingredient in pesto, basil, is considered a source of bioactive compounds, primarily phenolic acids and flavonoids, which contribute to its strong antioxidant, health-promoting, and sensory properties. However, regarding the content of total phenols in basil, the literature values differ significantly (even by an order of magnitude). This variability may be due to differences in the method of cultivation, the time of harvesting the plant, its variety, the method of storage, and the methodological approaches adopted [42,43]. The literature found data on TPC in basil pesto similar to those in the presented work. De Bruno et al. [1], depending on the variant of pesto prepared (fresh, pasteurized, with the addition of natural antioxidant extract, and after storage time), found TPC in it ranging from 1.0 to 2.06 mg GEA/g. However, attention should also be paid to the pesto recipe itself, as the traditional recipe was modified in the work presented here, which may also affect the bioactive content of the product. Nevertheless, it was unequivocally found that adding spirulina to basil pesto increased the sauce’s polyphenols, flavonoids, and antioxidant activity. Recently, much attention has been paid to the possible role of foods fortified with bioactive compounds from microalgae, due to their safety and efficacy in treating many diseases. The antioxidant activity of microalgae is a result of their abundance of compounds that can scavenge free radicals [11], including the aforementioned phenolic compounds, flavonoids, or carotenoids, and the blue pigment phycocyanin found in spirulina, which has antioxidant potential in addition to imparting color [4]. As a high-protein product, spirulina is also a potential source of bioactive proteins and peptides. Several peptides derived from spirulina with *in vitro* or *in vivo* antioxidant activity have been described [37]. Many authors have observed an increase in antioxidant compounds and antioxidant activity in foods after spirulina enrichment, including pasta [8], bread [10], ice cream [44], Ricotta cheese [13], and vegan kefir [5], among others. 

Due to its properties, the resulting pesto with spirulina is competitive with its traditional counterparts on store shelves. Consumer research, which is extremely important in the design of functional foods, has also confirmed this. It was concerned that a product with the addition of marine algae might be less acceptable to the consumer due to the marked change in color of the sauce and the characteristic taste of spirulina. However, the results of the semi-consumer evaluation prove that adding spirulina in appropriate amounts can contribute to the attractiveness of basil sauce. Other researchers have obtained similar results. For example, Barakat et al. [45] confirmed that adding spirulina up to 2% to cookies did not significantly affect their taste, aroma, and texture. For higher additions of algae, the results of sensory evaluation and overall acceptability of the cookies significantly decreased. Similarly, in the case of spirulina-enriched chicken mortadella, the best flavor and overall rating, according to respondents, was the product enriched with 2% and 3% algae [17].

An important quality feature for industrial processing and the final product, which strongly affects consumer choice, is the characteristic green color of pesto sauce [42,46]. The chlorophylls (chlorophyll a and b) found in basil are primarily responsible for this attribute. Unfortunately, these pigments are especially sensitive to many factors, including pH, temperature, or the presence of enzymes, which can modify the original color by transforming chlorophylls into the corresponding brownish pheophytins [46]. Phenolic oxidation and enzymatic and non-enzymatic browning can also occur during the processing and storage of pesto sauce [42]. This all leads to a change in the color of the sauce, reducing the intensity of the green, and thus the product’s attractiveness. The study assumed that adding intense green spirulina to basil pesto would affect the final product’s color. As expected, a darker sauce was obtained with a clear, noticeable difference between the control samples. However, this did not negatively affect the product quality; on the contrary, consumers rated the color of the sauce with 1% spirulina added as the highest. Product quality characteristics largely determine texture parameters and often correlate with the sensory evaluation of indicators such as texture and structure [47]. Thus, determining texture parameters was an essential part of sauce design. In the study, differences were noted only in hardness between the sauce with spirulina and the control pesto. The sample with the 2% addition had the highest hardness, which resulted in the lowest consistency score in the consumer evaluation. The available scientific literature has observed no clear upward or downward trend in texture parameters with increasing spirulina addition. For example, in the Boyanova et al. [47] study, ice cream with spirulina had a lower hardness than the control ice cream. In contrast, Ismail et al. [13] found that spirulina-enriched Ricotta cheese was harder than its non-algae-enriched counterpart. In turn, Batista et al. [48] found no significant differences in the hardness of crackers with spirulina added (2% and 6%) compared to control crackers.

## 5. Conclusions

The designed vegan pesto with 1% and 2% added spirulina is a highly competitive product to traditional basil pesto. This consists of increased protein content (35% and 40% higher, respectively), minerals (including up to three times more iron and 25% more calcium), phenolic compounds (up to 50% more), and flavonoids (up to 39% more), higher antioxidant activity against DPPH (up to 392.41 ± 13.58 μM Trolox/g) and ABTS (up to 484.56 ± 2.16 μM Trolox/g), with high consumer scores (4.56 and 4.01). The results obtained may have potential applications and be helpful, especially for producers of ready-to-eat sauces. Due to the busy lifestyles of many consumers, health-promoting, quick-to-prepare products are extremely popular. In addition, the growing number of vegans and vegetarians is prompting the development of new, competitive products aimed at this group.

## Figures and Tables

**Table 1:** Basil pesto recipe.

Sample	Grape Seed Oil	Basil	Cashew Nuts	Yeast Flakes	Lemon Juice	Salt	Spirulina	Granulated Garlic
	[g/100 g]							
BP0	35	31	25	5.7	2	1	-	0.3
BP1	35	30	25	5.7	2	1	1	0.3
BP2	35	29	25	5.7	2	1	2	0.3

BP0—control basil pesto, BP1—basil pesto with 1% addition of spirulina, BP2—basil pesto with 2% addition of spirulina.

**Table 2:** Color determinants of tested basil pesto.

Sample	L*	a*	b*	ΔE
BP0	43.03 ± 0.58 a *	−5.75 ± 0.33 a	19.57 ± 0.32 a	-
BP1	36.92 ± 0.39 b	−5.79 ± 0.24 a	13.19 ± 0.26 b	8.83
BP2	34.77 ± 0.83 c	−5.99 ± 0.24 a	10.80 ± 0.19 c	12.05

BP0—control basil pesto, BP1—basil pesto with 1% addition of spirulina, BP2—basil pesto with 2% addition of spirulina. * In each column, results with the same letter are not statistically significantly different at *p* < 0.05 (Tukey’s post hoc test).

**Table 3:** Nutritional value of tested basil pesto.

Sample	Protein	Carbohydrates	Fat	Ash	Moisture	Energy Value [kcal/100 g]
	g/100 g Fresh Pesto					
BP0	6.47 ± 0.25 b *	9.89 ± 0.47 a	39.32 ± 0.17 b	1.83 ± 0.10 a	43.02 ± 1.14 a	419.33 ± 4.42 a
BP1	8.73 ± 0.38 a	6.67 ± 0.52 b	40.46 ± 0.13 a	1.99 ± 0.09 a	42.21 ± 0.92 a	425.72 ± 0.03 a
BP2	9.13 ± 0.20 a	6.97 ± 0.43 b	40.43 ± 0.21 a	1.75 ± 0.02 a	41.72 ± 0.85 a	428.26 ± 0.99 a

BP0—control basil pesto, BP1—basil pesto with 1% addition of spirulina, BP2—basil pesto with 2% addition of spirulina. * In each column, results with the same letter are not statistically significantly different at *p* < 0.05 (Tukey’s post hoc test).

**Table 4:** *In vitro* protein digestibility and protein availability.

Sample	PD [%]	PA [g/100 g]
BP0	79.89 ± 0.33 a *	5.17 ± 0.22 b
BP1	78.75 ± 0.12 a	6.88 ± 0.29 a
BP2	77.88 ± 1.12 a	7.11 ± 0.02 a

BP0—control basil pesto, BP1—basil pesto with 1% addition of spirulina, BP2—basil pesto with 2% addition of spirulina, PD—*in vitro* protein digestibility, PA—protein availability. * In each column, results with the same letter are not statistically significantly different at *p* < 0.05 (Tukey’s post hoc test).

**Table 5:** Mineral content of tested basil pesto.

Sample	Ca	Mg	K	Fe	P
	mg/100 g Fresh Pesto				
BP0	78.66 ± 2.88 b *	81.14 ± 2.68 b	342.32 ± 6.18 c	1.98 ± 0.09 c	177.71 ± 3.12 b
BP1	97.64 ± 0.65 a	87.04 ± 2.63 ab	382.45 ± 4.85 b	4.09 ± 0.12 b	185.51 ± 5.26 b
BP2	98.47 ± 3.84 a	91.63 ± 0.44 a	408.77 ± 2.18 a	6.15 ± 0.07 a	234.46 ± 2.62 a

BP0—control basil pesto, BP1—basil pesto with 1% addition of spirulina, BP2—basil pesto with 2% addition of spirulina. * In each column, results with the same letter are not statistically significantly different at *p* < 0.05 (Tukey’s post hoc test).

**Table 6:** Bioactive properties of tested basil pesto.

Sample	TPC [mg GAE/100 g]	TFC [mg EPI/100 g]	DPPH [μM Trolox/g]	ABTS [μM Trolox/g]
BP0	172.96 ± 4.6 c *	21.50 ± 0.13 c	290.97 ± 14.71 c	439.61 ± 9.76 c
BP1	206.32 ± 6.34 b	24.82 ± 0.28 b	353.66 ± 5.11 b	459.00 ± 6.68 b
BP2	259.24 ± 9.30 a	29.88 ± 0.8 a	392.41 ± 13.58 a	484.56 ± 2.16 a

BP0—control basil pesto, BP1—basil pesto with 1% addition of spirulina, BP2—basil pesto with 2% addition of spirulina. * In each column, results with the same letter are not statistically significantly different at *p* < 0.05 (Tukey’s post hoc test).

**Table 7:** Texture parameter results of the tested basil pesto.

Sample	Hardness [N]	Adhesiveness	Cohesion	Springiness
BP0	70.46 ± 1.50 c *	−47.92 ± 1.28 a	0.08 ± 0.01 a	13.28 ± 0.42 a
BP1	76.31 ± 2.25 b	−53.87 ± 2.87 a	0.08 ± 0.01 a	12.90 ± 0.49 a
BP2	83.62 ± 1.14 a	−51.03 ± 5.24 a	0.08 ± 0.01 a	13.04 ± 0.40 a

BP0—control basil pesto, BP1—basil pesto with 1% addition of spirulina, BP2—basil pesto with 2% addition of spirulina. * In each column, results with the same letter are not statistically significantly different at *p* < 0.05 (Tukey’s post hoc test).

**Table 8:** Semi-consumer evaluation of the tested basil pesto.

Sample	Evaluation Parameters					Overall Rating
	Overall Appearance	Color	Taste	Aroma	Consistency	
BP0	4.26 ± 0.70 b *	4.09 ± 0.78 b	4.17 ± 0.71 b	4.23 ± 0.60 a	4.34 ± 0.76 ab	4.22
BP1	4.77 ± 0.43 a	4.49 ± 0.61 a	4.57 ± 0.50 a	4.26 ± 0.61 a	4.49 ± 0.66 a	4.56
BP2	4.00 ± 0.64 b	3.97 ± 0.62 b	4.00 ± 0.69 b	4.09 ± 0.61 a	4.03 ± 0.66 b	4.01

BP0—control basil pesto, BP1—basil pesto with 1% addition of spirulina, BP2—basil pesto with 2% addition of spirulina. * In each column, results with the same letter are not statistically significantly different at *p* < 0.05 (Tukey’s post hoc test).

## Data Availability

The original contributions presented in the study are included in the article; further inquiries can be directed to the corresponding authors.

## References

[1] De Bruno: A., Gattuso A., Romeo R., Santacaterina S., Piscopo A. (2022). Functional and Sustainable Application of Natural Antioxidant Extract Recovered from Olive Mill Wastewater on Shelf-Life Extension of “Basil Pesto”. Appl. Sci..

[2] Durazzo A., Camilli E., Marconi S., Lisciani S., Gabrielli P., Gambelli L., Aguzzi A., Lucarini M., Kiefer J., Marletta L. (2019). Nutritional Composition and Dietary Intake of Composite Dishes Traditionally Consumed in Italy. J. Food Compos. Anal..

[3] Turini E., Sarsale M., Petri D., Totaro M., Lucenteforte E., Tavoschi L., Baggiani A. (2022). Efficacy of Plant Sterol-Enriched Food for Primary Prevention and Treatment of Hypercholesterolemia: A Systematic Literature Review. Foods.

[4] Bortolini D.G., Maciel G.M., Fernandes I.d.A.A., Pedro A.C., Rubio F.T.V., Branco I.G., Haminiuk C.W.I. (2022). Functional Properties of Bioactive Compounds from *Spirulina* spp.: Current Status and Future Trends. Food Chem. Mol. Sci..

[5] Sözeri Atik D., Gürbüz B., Bölük E., Palabıyık İ. (2021). Development of Vegan Kefir Fortified with *Spirulina platensis*. Food Biosci..

[6] Janda-Milczarek K., Szymczykowska K., Jakubczyk K., Kupnicka P., Skonieczna-Żydecka K., Pilarczyk B., Tomza-Marciniak A., Ligenza A., Stachowska E., Dalewski B. (2023). Spirulina Supplements as a Source of Mineral Nutrients in the Daily Diet. Appl. Sci..

[7] Trotta T., Porro C., Cianciulli A., Panaro M.A. (2022). Beneficial Effects of Spirulina Consumption on Brain Health. Nutrients.

[8] Hussein A., Ibrahim G., Kamil M., El-Shamarka M., Mostafa S., Mohamed D. (2021). Spirulina-Enriched Pasta as Functional Food Rich in Protein and Antioxidant. Biointerface Res. Appl. Chem..

[9] Rodríguez De Marco E., Steffolani M.E., Martínez C.S., León A.E. (2014). Effects of Spirulina Biomass on the Technological and Nutritional Quality of Bread Wheat Pasta. LWT-Food Sci. Technol..

[10] Hernández-López I., Alamprese C., Cappa C., Prieto-Santiago V., Abadias M., Aguiló-Aguayo I. (2023). Effect of Spirulina in Bread Formulated with Wheat Flours of Different Alveograph Strength. Foods.

[11] Barkallah M., Dammak M., Louati I., Hentati F., Hadrich B., Mechichi T., Ayadi M.A., Fendri I., Attia H., Abdelkafi S. (2017). Effect of *Spirulina platensis* Fortification on Physicochemical, Textural, Antioxidant and Sensory Properties of Yogurt During Fermentation and Storage. LWT-Food Sci. Technol..

[12] Çelekli A., Alslibi Z.A., Bozkurt H. (2019). Üseyin Influence of Incorporated *Spirulina Platensis* on the Growth of Microflora and Physicochemical Properties of Ayran as a Functional Food. Algal Res..

[13] Ismail H.A., El-Sawah T.H., Ayyash M., Adhikari B., Elkot W.F. (2023). Functionalization of Ricotta Cheese with Powder of *Spirulina platensis*: Physicochemical, Sensory, and Microbiological Properties. Int. J. Food Prop..

[14] Szmejda K., Duliński R., Byczyński Ł., Karbowski A., Florczak T., Żyła K. (2018). Analysis of the Selected Antioxidant Compounds in Ice Cream Supplemented with Spirulina (*Arthrospira platensis*) Extract. Biotechnol. Food Sci..

[15] Paula da Silva S., Ferreira do Valle A., Perrone D. (2021). Microencapsulated Spirulina Maxima Biomass as an Ingredient for the Production of Nutritionally Enriched and Sensorially Well-Accepted Vegan Biscuits. LWT-Food Sci. Technol..

[16] Batista de Oliveira T.T., Miranda dos Reis I., Bastos de Souza M., da Silva Bispo E., Fonseca Maciel L., Druzian J.I., Lordelo Guimarães Tavares P.P., de Oliveira Cerqueira A., dos Santos Boa Morte E., Abreu Glória M.B. (2021). Microencapsulation of *Spirulina* Sp. LEB-18 and Its Incorporation in Chocolate Milk: Properties and Functional Potential. LWT-Food Sci. Technol..

[17] El-Anany A.M., Althwab S.A., Alhomaid R.M., Ali R.F.M., Mousa H.M. (2023). Effect of Spirulina (*Arthrospira platensis*) Powder Addition on Nutritional and Sensory Attributes of Chicken Mortadella. Ital. J. Food Sci..

[18] Gromek W., Kołdej N., Kurowski M., Majsiak E. (2024). Spirulina (*Arthrospira platensis*): Antiallergic Agent or Hidden Allergen? A Literature Review. Foods.

[19] Prete V., Abate A.C., Di Pietro P., De Lucia M., Vecchione C., Carrizzo A. (2024). Beneficial Effects of Spirulina Supplementation in the Management of Cardiovascular Diseases. Nutrients.

[20] Torres-Duran P.V., Ferreira-Hermosillo A., Juarez-Oropeza M.A. (2007). Antihyperlipemic and Antihypertensive Effects of Spirulina Maxima in an Open Sample of Mexican Population: A Preliminary Report. Lipids Health Dis..

[21] Lafarga T., Fernández-Sevilla J.M., González-López C., Acién-Fernández F.G. (2020). Spirulina for the Food and Functional Food Industries. Food Res. Int..

[22] AOAC International (2010). Official Methods of Analysis of AOAC International.

[23] Fogarasi M., Urs M.J., Socaciu M.I., Ranga F., Semeniuc C.A., Vodnar D.C., Mureșan V., Țibulcă D., Fogarasi S., Socaciu C. (2024). Polyphenols-Enrichment of Vienna Sausages Using Microcapsules Containing Acidic Aqueous Extract of Boletus Edulis Mushrooms. Foods.

[24] Zielińska E., Karaś M., Jakubczyk A. (2017). Antioxidant Activity of Predigested Protein Obtained From a Range of Farmed Edible Insects. Int. J. Food Sci. Technol..

[25] Jorhem L., Engman J., Collaborators, Arvidsson B.-M., Åsman B., Åstrand C., Gjerstad K.O., Haugsnes J., Heldal V., Holm K. (2000). Determination of Lead, Cadmium, Zinc, Copper, and Iron in Foods by Atomic Absorption Spectrometry after Microwave Digestion: NMKL1 Collaborative Study. J. AOAC Int..

[26] Implementing Regulation-EU-2024/771-EN-EUR-Lex. https://eur-lex.europa.eu/eli/reg_impl/2024/771/oj.

[27] Karadeniz F., Burdurlu H.S., Koca N., Soyer Y. (2005). Antioxidant Activity of Selected Fruits and Vegetables Grown in Turkey. Turk. J. Agric. For..

[28] Baliyan S., Mukherjee R., Priyadarshini A., Vibhuti A., Gupta A., Pandey R.P., Chang C.M. (2022). Determination of Antioxidants by DPPH Radical Scavenging Activity and Quantitative Phytochemical Analysis of *Ficus religiosa*. Molecules.

[29] Re R., Pellegrini N., Proteggente A., Pannala A., Yang M., Rice-Evans C. (1999). Antioxidant Activity Applying an Improved ABTS Radical Cation Decolorization Assay. Free Radic. Biol. Med..

[30] Szafrańska J., Muszyński S., Sołowiej B. (2019). Ocena Właściwości Fizykochemicznych Sosów Serowych Otrzymanych Na Bazie Kazeiny Kwasowej i Oleju Rzepakowego z Dodatkiem Koncentratu Białek Serwatkowych. Przemysł Spożywczy.

[31] Almohtadi R.M., Aldarabah I.T. (2021). University Students’ Attitudes toward the Formal Integration of Facebook in Their Education: Investigation Guided by Rogers’ Attributes of Innovation. World J. Educ..

[32] Arwanto V., Buschle-Diller G., Mukti Y.P., Dewi A.D.R., Mumpuni C., Purwanto M.G.M., Sukweenadhi J. (2022). The State of Plant-Based Food Development and Its Prospects in the Indonesia Market. Heliyon.

[33] Hassoun A., Cropotova J., Trif M., Rusu A.V., Bobiş O., Nayik G.A., Jagdale Y.D., Saeed F., Afzaal M., Mostashari P. (2022). Consumer Acceptance of New Food Trends Resulting From the Fourth Industrial Revolution Technologies: A Narrative Review of Literature and Future Perspectives. Front. Nutr..

[34] Morsy O.M., Sharoba A.M., El-Desouky A.I., Bahlol HE M., Abd El Mawla E.M. (2014). Production and Evaluation of Some Extruded Food Products Using Spirulina Algae. Ann. Agric. Sci. Moshtohor.

[35] Carnauba R.A., Baptistella A.B., Paschoal V., Hübscher G.H. (2017). Diet-Induced Low-Grade Metabolic Acidosis and Clinical Outcomes: A Review. Nutrients.

[36] Lupatini A.L., Colla L.M., Canan C., Colla E. (2017). Potential Application of Microalga *Spirulina Platensis* as a Protein Source. J. Sci. Food Agric..

[37] Lafarga T., Sánchez-Zurano A., Villaró S., Morillas-España A., Acién G. (2021). Industrial Production of Spirulina as a Protein Source for Bioactive Peptide Generation. Trends Food Sci. Technol..

[38] Bueschke M., Gramza-Michałowska A., Kubiak T., Kulczyński B. (2017). Alternatywne Źródła Białka w Żywieniu Człowieka. Zesz. Nauk. SGGW Warszawie-Probl. Rol. Swiat..

[39] Ramírez-Rodrigues M.M., Estrada-Beristain C., Metri-Ojeda J., Pérez-Alva A., Baigts-Allende D.K. (2021). *Spirulina Platensis* Protein as Sustainable Ingredient for Nutritional Food Products Development. Sustainability.

[40] Cader P., Lesiów T. (2021). Weganizm i Wegetarianizm Jako Diety We Współczesnym Społeczeństwie Konsumpcyjnym. Nauk. Inżynierskie Technol..

[41] Kumar S.B., Arnipalli S.R., Mehta P., Carrau S., Ziouzenkova O. (2022). Iron Deficiency Anemia: Efficacy and Limitations of Nutritional and Comprehensive Mitigation Strategies. Nutrients.

[42] Sordini B., Urbani S., Esposto S., Selvaggini R., Daidone L., Veneziani G., Servili M., Taticchi A. (2024). Evaluation of the Effect of an Olive Phenolic Extract on the Secondary Shelf Life of a Fresh Pesto. Antioxidants.

[43] Prinsi B., Morgutti S., Negrini N., Faoro F., Espen L. (2019). Insight into Composition of Bioactive Phenolic Compounds in Leaves and Flowers of Green and Purple Basil. Plants.

[44] Bürck M., Fratelli C., Assis M., Braga A.R.C. (2024). Naturally Colored Ice Creams Enriched with C-Phycocyanin and Spirulina Residual Biomass: Development of a Fermented, Antioxidant, Tasty and Stable Food Product. Fermentation.

[45] Barakat E.H., El-Kewaisny N.M., Salama A.A. (2016). Chemical and Nutritional Evaluation of Fortified Biscuits with Dried Spirulina Algae. J. Food Dairy Sci..

[46] Turrini F., Farinini E., Leardi R., Grasso F., Orlandi V., Boggia R. (2022). A Preliminary Color Study of Different Basil-Based Semi-Finished Products during Their Storage. Molecules.

[47] Boyanova P., Gradinarska D., Dobreva V., Panayotov P., Momchilova M., Zsivanovits G. (2022). Effect of *Spirulina platensis* on the Quality and Antioxidants Characteristics of Ice Cream. BIO Web Conf..

[48] Batista A.P., Niccolai A., Bursic I., Sousa I., Raymundo A., Rodolfi L., Biondi N., Tredici M.R. (2019). Microalgae as Functional Ingredients in Savory Food Products: Application to Wheat Crackers. Foods.

